# Editorial for the Topic on Micromachining for Advanced Biological Imaging

**DOI:** 10.3390/mi13030474

**Published:** 2022-03-19

**Authors:** Young Jin Yoo, Young Min Song

**Affiliations:** 1 School of Electrical Engineering and Computer Science, Gwangju Institute of Science and Technology, 123 Cheomdangwagi-ro, Buk-gu, Gwangju 61005, Korea; yjyoo89@gist.ac.kr; 2 AI Graduate School, Gwangju Institute of Science and Technology, 123 Cheomdangwagi-ro, Buk-gu, Gwangju 61005, Korea

Biological imaging has opened novel paths for discoveries and advances in biology ranging from molecular to tissue scales. It involves techniques and methods that are based on imaging approaches, especially quantitative and computational imaging. It uses light, fluorescence, electrons, ultrasound, X-ray, magnetic resonance, and positrons as sources for imaging. Bioimaging aims to interfere as little as possible with life processes, enabling the non-invasive visualization/analysis of biological processes in real life. Recent developments in bioimaging include many different types of advanced imaging methods, such as super-resolved imaging, three-dimensional imaging, two-photon fluorescence excitation microscopy, fluorescence resonance energy transfer (FRET), and stochastic optical reconstruction microscopy (STORM). Such efforts have resulted in several Nobel Prizes being awarded to scientists who have made remarkable contributions.

Despite such tremendous achievements, the quest for high-resolution bioimaging and quantitative analysis is far from over and still faces many challenges. Micromachining has been developed in research and industrial environments to improve the quality of imaging of biological samples. Today, various types of micro- and nano-structures play important roles in advanced bioimaging applications. Accordingly, in this Editorial, we highlight reviews and original research articles that focus on the use of micromachining for advanced biological imaging, including in micro-optics [1,2,3], nanopatterning [4,5], and optical imaging with microfluidics [6,7,8].

The 3D imaging of a biological sample provides invaluable information regarding cell biology and the pathology of related diseases. However, the currently available 3D imaging tools are complex and expensive. Lee et al. report the use of a micromirror-embedded coverslip assembly for bidirectional microscopic imaging [1]. Microfabricated mirrors with a high reflectivity enable bidirectional imaging with the simple control of a focal plane. The device provides a quick analysis of 3D positions and subcellular structures. In some imaging applications, microlens arrays (MLAs) have become indispensable optical components due to their interesting optic characteristics and simple configurations. However, their narrow depth-of-field remains a challenge for reliable depth estimation. Lee et al. report a novel wafer-level fabrication method for use in multifocal MLAs with a steady and high NA through one-step lithography [2]. They also demonstrate the multi-focal plane image acquisition via multi-focal MLAs integrated in a microscope. Imaging tools with a mid-infrared (MIR) range can provide a broad insight into cells and tissues. Lee et al. report design and fabrication schemes for microscale silicon solid immersion lenses based on thin-film geometry for mid-infrared applications [3]. The fabricated silicon lenses provide outstanding near-field focusing, which corresponds to a high numerical aperture.

Optic polarizers and spectral filters enable contrast enhancement for biological specimens. Since chiral structures have different dielectric constants for right circularly polarized and left circularly polarized light, they exhibit circular dichroism, which affords a strong image contrast. Furusawa et al. present a novel method for generating circular dichroism based on Au nanospirals transferred onto PDMS film [4]. The fabricated sample shows a very large circular dichroism peak at visible wavelengths. Chang et al. review various types of spectral color filters based on vertically aligned nanowires for visible and near-infrared ranges [5]. This review article focuses on the core optical properties of nanowires, fabrication methods, and examples of photonic applications.

A wide variety of detection options in macro-scale optical infrastructure can be coupled to microfluidic devices. Zhou et al. review an optical imaging system combined with microfluidics, including bright-field microscopy, spectrum-based microscopy imaging, and fluorescence-based microscopy imaging [6]. Droplet microfluidics involves chemicals or cellular reagents that can be used for large-scale chemical or biological analysis. Dobson et al. propose a method for photo-tagging individual microfluidic droplets for latter selection by passive sorting [7]. This is a simple and inexpensive method that can be performed on a conventional fluorescence microscope that uncouples the observation and selection of droplets. Youssef et al. report the development of an easy-to-use microfluidic electrotaxis-based chip that can be used to investigate the behavior and neuron degeneration of 16 worms in parallel [8]. They show its applicability for genetic, chemical, and neuronal screening after validating it against a single-worm electrotaxis assay.
